# Draft genome sequence of *Bacillus thuringiensis* GIFSPR-111, a putative probiotic isolated from shrimp farm soil in Bangladesh

**DOI:** 10.1128/mra.00277-26

**Published:** 2026-08-05

**Authors:** Md. Rony Babu, Promi Saha, Santu Biswas, Arpita Guha, Farzana Yeasmin, Tasmina Akter, Dipali Rani Gupta, Tofazzal Islam, Md. Mahbubur Rahman

**Affiliations:** 1 Institute of Biotechnology and Genetic Engineering, Gazipur Agricultural University 198780, Gazipur, Bangladesh; 2 Department of Fisheries Management, Gazipur Agricultural University 198780, Gazipur, Bangladesh; Montana State University, Bozeman, Montana, USA

**Keywords:** *Bacillus thuringiensis*, shrimp probiotic, *Penaeus monodon*, aquaculture, bioactive compound

## Abstract

*Bacillus thuringiensis* GIFSPR-111, isolated from shrimp farm soil in Shamnagar, Bangladesh, inhibits *Vibrio parahaemolyticus*, the causative agent of acute hepatopancreatic necrosis disease in shrimp and exhibits probiotic potential. The 5,608,442 bp draft genome contains multiple biosynthetic gene clusters, with potential to synthesize diverse bioactive metabolites.

## ANNOUNCEMENT

Members of the genus *Bacillus* are widely used in aquaculture as probiotics because of their beneficial effects on host growth, immunity, and disease resistance (1–4). In this study, *Bacillus thuringiensis* strain GIFSPR-111 was isolated from shrimp farm soil in Shamnagar (22°16′08.0″N 89°12′42.1″E), Satkhira, Bangladesh.

Soil samples were serially diluted and cultured on de Man, Rogosa, and Sharpe (MRS) agar at 28°C for 48 h. A single colony was purified through repeated streaking. Although MRS agar is commonly used for lactic acid bacteria, it can also support the growth of other gram-positive bacteria, including *Bacillus* spp., during initial screening. Taxonomic identification was initially performed by 16S rRNA gene sequencing, and the sequence was deposited in the NCBI GenBank database under accession number PX850636.1. Whole-genome analysis and annotation using the NCBI Prokaryotic Genome Annotation Pipeline (PGAP) v6.10 (5) further confirmed the identity of the strain.

**Fig 1:**
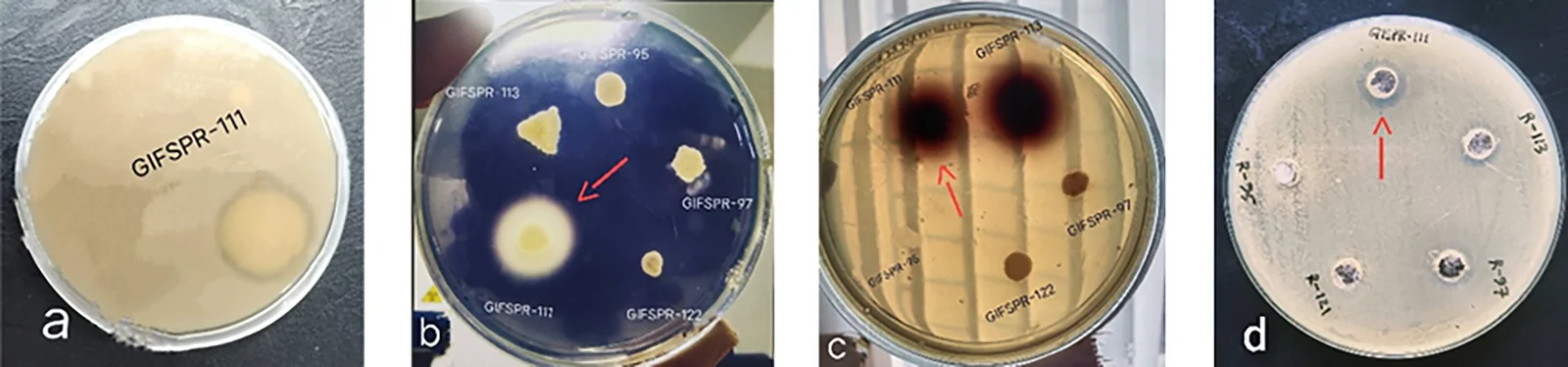
Extracellular enzymatic and antagonistic activities of *Bacillus thuringiensis* GIFSPR-111. Petri dishes demonstrate (**a**) Protease on skim milk agar, confirmed by a transparent zone. (**b**) Amylase on starch agar, indicated by a clear halo after iodine staining. (**c**) Bile esculin hydrolysis and tolerance, indicated by blackening of medium. (**d**) Antagonistic activity against *V. parahaemolyticus* GISV1-1, showing a clear zone of inhibition.

*In vitro* probiotic characteristics of GIFSPR-111 were evaluated, including protease activity on 5% skim milk agar (6), amylase activity on starch agar (6), bile salt tolerance and hydrolysis on bile esculin-supplemented medium (7), and antimicrobial activity using the agar well diffusion method (8). The strain exhibited protease, amylase, bile salt tolerance and esculin hydrolysis, and antagonistic activities against *Vibrio parahaemolyticus* strain GISV1-1 (GenBank accession number NZ_JBSZAZ000000000.1) (Fig. 1).

For genome sequencing, the strain was cultured in Todd Hewitt Broth at 37°C. Genomic DNA was extracted using the DNeasy Blood & Tissue Kit (Qiagen). DNA quality and quantity were assessed using NanoDrop spectrophotometer, Qubit fluorometer (Thermo Fisher Scientific, USA), and LabChip GX Bioanalyzer (PerkinElmer). Sequencing libraries were prepared using the Illumina DNA Prep Kit and sequenced on an Illumina NextSeq 2000 platform, generating 2 × 150 bp paired-end reads.

A total of 961,225 paired-end reads were obtained, providing approximately 49× genome coverage. Raw reads were quality checked using FastQC v0.12.1 (9) and trimmed using Trimmomatic v0.39 (10). *De novo* assembly was performed using SPAdes v4.2.0 (11). Genome completeness was assessed using CheckM v1.2.4 (12), which showed 98.51% completeness and 0.65% contamination.

The draft genome assembly comprises 5,608,442 bp distributed across 118 contigs, with a GC content of 35%. The N50 and L50 values are 211,301 bp and 8, respectively. Genome annotations using PGAP v6.10 identified a total of 5,823 genes, including 5,737 coding sequences, 69 tRNAs, 11 rRNAs, 5 non-coding RNAs, and 190 pseudogenes. Functional annotation using RAST (13) identified 338 subsystems.

Genome analysis revealed the presence of a fosfomycin resistance gene (*fosB1*), identified using ResFinder v4.7.2 (14). No plasmid replicons or CRISPR arrays were detected using PlasmidFinder v2.0 (15) and CRISPRCasFinder (16), respectively. AntiSMASH v6.0 (17) analysis identified 13 biosynthetic gene clusters, including non-ribosomal peptide synthetases, ribosomally synthesized and post-translationally modified peptides, terpenes, and type II polyketide biosynthesis gene cluster, suggesting the potential for production of diverse bioactive metabolites.

The genome sequence of *Bacillus thuringiensis* GIFSPR-111 provides valuable insights into its genetic and probiotic characteristics, supporting its potential application in shrimp aquaculture.

## Data Availability

The whole-genome shotgun project of *Bacillus thuringiensis* GIFSPR-111 has been deposited at GenBank under the accession number JBSZAY000000000. The associated BioProject and BioSample accession numbers are PRJNA1378491 and SAMN53839583, respectively. The raw data are available from the Sequence Read Archive under accession number SRX31557332. The 16S rRNA gene sequence is available under accession number PX850636.1.
